# Circular RNAs in breast cancer diagnosis, treatment and prognosis

**DOI:** 10.32604/or.2023.046582

**Published:** 2023-12-28

**Authors:** XIAOJIA HUANG, CAILU SONG, JINHUI ZHANG, LEWEI ZHU, HAILIN TANG

**Affiliations:** 1 Department of Breast Oncology Surgery, Affiliated Cancer Hospital & Institute of Guangzhou Medical University, Guangzhou, 510095, China; 2 State Key Laboratory of Oncology in South China, Guangdong Provincial Clinical Research Center for Cancer, Sun Yat-sen University Cancer Center, Guangzhou, 510060, China; 3 Department of Breast Surgery, The First People’s Hospital of Foshan, Foshan, 528000, China

**Keywords:** CircRNA, Breast cancer, Diagnosis, Treatment, Biomarker

## Abstract

Breast cancer has surpassed lung cancer to become the most common malignancy worldwide. The incidence rate and mortality rate of breast cancer continue to rise, which leads to a great burden on public health. Circular RNAs (circRNAs), a new class of noncoding RNAs (ncRNAs), have been recognized as important oncogenes or suppressors in regulating cancer initiation and progression. In breast cancer, circRNAs have significant roles in tumorigenesis, recurrence and multidrug resistance that are mediated by various mechanisms. Therefore, circRNAs may serve as promising targets of therapeutic strategies for breast cancer management. This study reviews the most recent studies about the biosynthesis and characteristics of circRNAs in diagnosis, treatment and prognosis evaluation, as well as the value of circRNAs in clinical applications as biomarkers or therapeutic targets in breast cancer. Understanding the mechanisms by which circRNAs function could help transform basic research into clinical applications and facilitate the development of novel circRNA-based therapeutic strategies for breast cancer treatment.

## Introduction

Breast cancer has surpassed lung cancer to become the most common malignancy worldwide, and its burden has continued to increase during the last decade. From 2010–2019, the incidence rate of breast cancer rose annually by 0.5% [1]. In 2020, over 2.3 million breast cancer cases were diagnosed, and approximately 685,000 related deaths occurred. By 2040, it is predicted that over 3 million breast cancer cases will occur every year. In transitioning countries, the incidence rate and mortality rate of breast cancer remain high and increase annually [2].

Noncoding RNAs (ncRNAs) play important roles as oncogenes or tumor suppressors in regulating cancer occurrence and development [3,4]. Many studies have indicated that ncRNAs have significant roles in breast cancer progression; therefore, ncRNAs may serve as promising therapeutics in breast cancer management [5,6]. Circular RNAs (circRNAs), a novel type of ncRNA, are synthesized via the back splicing of protein-coding genes. CircRNAs are involved in multiple biological processes and diseases [7]. CircRNAs are highly stable due to their covalently closed circular structures, which protects them from degradation by exonucleases and makes them promising candidate biomarkers for cancer diagnosis or prognosis evaluation [8].

**Figure 1:**
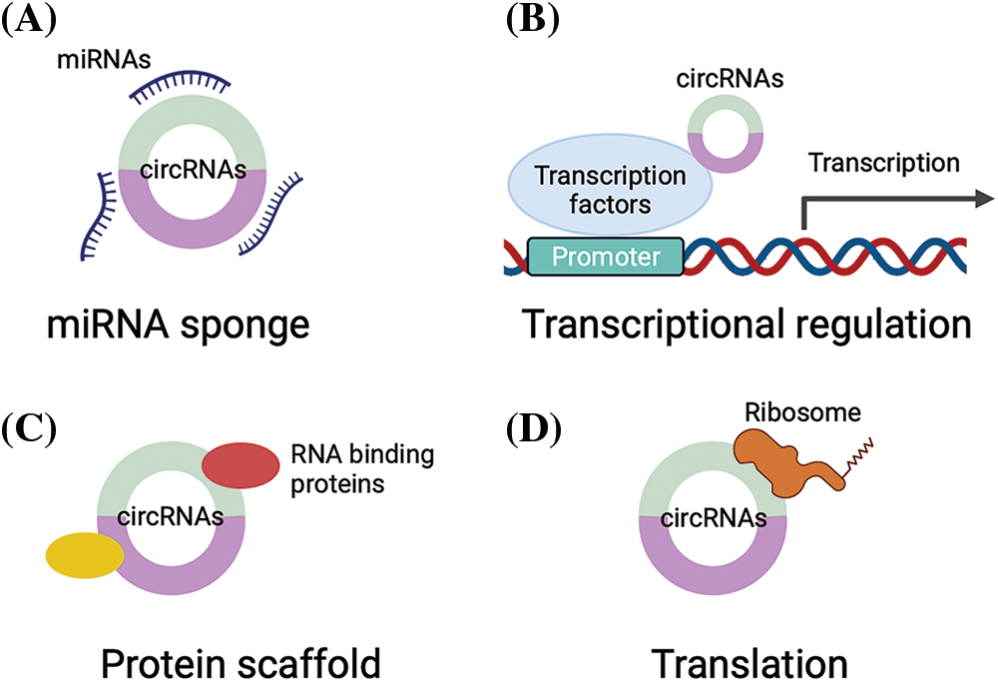
The regulatory mechanisms of circRNAs. (A) circRNAs can sponge miRNAs to suppress the activity of miRNAs; (B) circRNAs can bind to transcription factors to regulate gene transcription; (C) circRNAs can serve as protein scaffolds to interact with RNA binding proteins to modulate protein–protein interactions; (D) circRNAs can be translated into proteins.

The expression and function of circRNAs vary in different cancers; they modulate gene expression and cancer progression via a series of mechanisms. The most studied mechanism is the competitive endogenous RNA (ceRNA) mechanism, in which circRNAs act as microRNA (miRNA) sponges to prevent the degradation of targeted mRNAs by miRNAs (Fig. 1A), which are a group of short, conserved RNAs that can regulate the expression of targeted genes by binding to the 3′-untranslated region [9]. For example, circ_0086722 could sponge miR-339-5p to reverse the suppression of STAT5A to promote the progression of prostate cancer [10]. On the other hand, circRNAs can regulate transcription factors to bind to the promoters of targeted genes to modulate the expression of targeted genes (Fig. 1B). Moreover, circRNAs can serve as scaffolds to modulate protein–protein interactions (Fig. 1C) [11]. Some circRNAs even have translational potential [12,13] (Fig. 1D).

CircRNA dysregulation has been discussed in various malignancies, including breast cancer [14,15]. CircRNAs take part in the initiation and progression of breast cancer via a variety of mechanisms, acting as either oncogenes or tumor suppressors. The dysregulation of circRNAs in breast cancer is correlated with cell proliferation, apoptosis, autophagy, invasion, migration and treatment resistance. Here, we review the most recent studies on the biosynthesis and characteristics of circRNAs, the roles of circRNA in diagnosis, treatment and prognosis evaluation, and the value of circRNAs in clinical application as biomarkers or therapeutic targets in breast cancer.

## Main Text

### CircRNAs in breast cancer diagnosis

Cancer cells can release circRNAs into serum, saliva or urine, and these circRNAs could be detected as diagnostic biomarkers or prognostic indicators [16]. Recently, the functions of circRNAs in breast cancer diagnosis have been investigated in a series of studies [17]. To identify relevant studies, we searched the NCBI PubMed database with the keywords “((circular RNA[Title/Abstract]) OR (circRNA[Title/Abstract])) AND ((plasma[Title/Abstract]) OR (serum[Title/Abstract]) OR (blood[Title/Abstract])) AND (“Breast Neoplasms”[MeSH Terms]) NOT review[Publication Type]”, and we obtained 19 studies, among which 17 studies were included in this review after manual selection. The blood circSMARCA5 level was shown to be decreased in breast cancer patients compared to normal controls, revealing the potential value of circSMARCA5 as a biomarker candidate for breast cancer [18]. Hsa_circ_0104824 was found to be downregulated in breast cancer peripheral blood samples, supporting the diagnostic function of hsa_circ_0104824 for patients with breast cancer [19]. Circ_0000977 is downregulated in triple-negative breast cancer (TNBC) plasma samples, indicating the potential diagnostic value of circ_0000977 in TNBC [20]. Hsa_circ_0008673 is significantly upregulated in breast cancer plasma samples, which represents a novel class of biomarkers with great diagnostic value [21]. CircRASSF2, hsa_circ_0069094, hsa_circ_0079876, hsa_circ_0017650 and hsa_circ_0017526 were all upregulated in breast cancer patients [22,23]. CircBCBM1 is overexpressed in breast cancer plasma samples and could function as a potential diagnostic biomarker [24]. The level of circPRMT5 in serum samples is elevated in breast cancer patients in comparison with healthy volunteers [25]. Circ_0076611 could be released from TNBC cells to exosomes and was detectable in breast cancer patient serum [26]. Circ0088088 and circ00000751 were found to be upregulated in serum exosomes of breast cancer patients compared with those of healthy controls, while circ00005795 was found to be downregulated [27]. Additionally, circHIF1A and circPSMA1 could be released into exosomes and were upregulated in the plasma of breast cancer patients [28,29]. Circ_0000745, circ_0001531 and circ_0001640 were all elevated in whole blood samples of breast cancer patients, revealing great value in the diagnosis of breast cancer [30]. The level of hsa_circ_0001785 in plasma was reported to have better diagnostic accuracy than that of CEA or CA15-3 in breast cancer [31] and may serve as a promising diagnostic biomarker. Overall, circRNAs are promising, stably expressed biomarkers for breast cancer diagnosis or progression prediction [32] (Table 1).

**Table 1:** CircRNAs in breast cancer diagnosis

Name	Dysregulation	Source
CircSMARCA5 [18], circ_0104824 [19]	Downregulated	Blood
Circ_0000745, circ_0001531, circ_0001640 [30]	Upregulated	Blood
Circ_0000977 [20]	Downregulated	Plasma
Circ_0008673 [21], circ_0069094, circ_0079876, circ_0017650, circ_0017526 [23], circBCBM1 [24], circHIF1A [28], circPSMA1 [29], circ_0001785 [31]	Upregulated	Plasma
CircRASSF2 [22], circPRMT5 [25], circ_0076611 [26], circ0088088, circ0000751 [27]	Upregulated	Serum
Circ0005795 [27]	Downregulated	Serum

### CircRNAs in breast cancer treatment

Breast cancer is a heterogeneous malignancy with various therapeutic approaches based on the particular molecular subtype; treatments include chemotherapy, radiotherapy, endocrine therapy, targeted therapy and immunotherapy [33]. These therapeutic strategies have significantly improved the clinical outcome of breast cancer patients. However, the development of resistance to treatment is a great obstacle that needs further investigation [34]. Moreover, predictive biomarkers for therapy response are important approaches to selecting ideal patient populations and treatment strategies [35]. Recently, ncRNAs have been applied as promising approaches in cancer treatment [36]. RNA-based therapeutics, including circRNA therapies, are currently under evaluation in many clinical trials [37–39]. In breast cancer, circRNAs participate in regulating crucial pathways related to treatment resistance.

### Chemotherapy

Chemotherapy is one of the main therapeutic strategies for breast cancer. Chemoresistance is a great obstacle for breast cancer treatment. Understanding the mechanism of chemoresistance would help develop novel targets and coping strategies. CircRNAs take part in regulating chemoresistance in breast cancer [40] (Table 2). Thus, they may serve as biomarkers as well as therapeutic targets to restore the chemotherapy sensitivity of breast cancer [69,70]. Hsa_circ_0000199 is overexpressed in TNBC, and knockdown of hsa_circ_0000199 could induce chemosensitivity by modulating the levels of miR-206 and miR-613 to inhibit the PI3K/Akt/mTOR pathway [41]. Eighteen circRNAs were dysregulated in adriamycin (ADM)-resistant breast cancer, and hsa_circ_0006528 regulated resistance against ADM through the miR-7-5p-Raf1 axis [42]. Circ_0085495 is overexpressed in ADM-resistant breast cancer, and silencing circ_0085495 repressed ADM resistance by regulating miR-873-5p/integrin β1 signaling [43]. Circ_0044556 is upregulated in breast cancer and could promote cell resistance to ADM treatment by modulating miR‑145/NRAS signaling [44]. Inhibition of circ_0001667 in breast cancer could reduce ADM resistance through the miR-4458/NCOA3 axis [45]. CircATXN7 was found to be highly expressed in breast cancer, and overexpression of circATXN7 led to reduced breast cancer doxorubicin sensitivity by affecting miR-149-5p/HOXA11 signaling [46]. In doxorubicin-resistant TNBC cells, circ-CREIT was downregulated, while overexpression of circ-CREIT restored doxorubicin sensitivity in TNBC. Moreover, exosomes containing circ-CREIT could enhance the sensitivity to doxorubicin in TNBC. Mechanistically, circ-CREIT promoted PKR degradation through polyubiquitylation to activate the RACK1/MTK1 apoptosis pathway. Therefore, circ-CREIT might serve as a promising therapeutic strategy for chemoresistance in TNBC [47]. The level of circKDM4C is decreased in breast cancer, while overexpression of circKDM4C could suppress doxorubicin resistance via the miR-548p/PBLD axis [48]. In paclitaxel (PTX)-resistant breast cancer, circ_0069094 is highly expressed, and inhibiting circ_0069094 could restore sensitivity to PTX in breast cancer with PTX resistance by regulating the miR-136-5p/YWHAZ axis [49]. CircAMOTL1 could enhance breast cancer PTX resistance by regulating AKT signaling [50]. Overexpression of circDUSP1 could restore sensitivity to PTX in TNBC through acting as a decoy of miR-76 and increasing the level of DACT2 in TNBC [51]. Overexpression of circSMARCA5 in breast cancer cells could induce sensitivity to the cytotoxic drugs cisplatin (DDP) or bleomycin. Mechanistically, CircSMARCA5 suppressed SMARCA5 transcription, leading to decreased expression of SMARCA5 and enhanced sensitivity to chemotherapy [18]. CircUBAP2 is elevated in DDP-resistant TNBC and could promote resistance to DDP by regulating miR-300/ASF1B signaling as well as the PI3K/AKT/mTOR axis in TNBC [52]. CircFAT1 induced oxaliplatin resistance in breast cancer by modulating the miR-525-5p/SKA1 axis as well as Notch and Wnt signaling [53]. CircCDR1as is highly expressed in 5-fluorouracil (5-FU)-resistant breast cancer and can regulate the chemosensitivity of 5-FU by modulating the miR-7/CCNE1 pathway [54]. Collectively, the above studies indicated that circRNAs might act as promising clinical biomarkers and treatment targets in breast cancer patients with chemoresistance.

**Table 2:** CircRNAs in breast cancer treatment

Name	Treatment	Response
Circ_0000199 [41]	Cisplatin, adriamycin, paclitaxel, gemcitabine	Resistant
Circ_0006528 [42], circ_0085495 [43], circ_0044556 [44], circ_0001667 [45]	Adriamycin	Resistant
CircATXN7 [46]	Doxorubicin	Resistant
CircCREIT [47], circKDM4C [48]	Doxorubicin	Sensitive
Circ_0069094 [49], circAMOTL1 [50]	Paclitaxel	Resistant
CircDUSP1 [51]	Paclitaxel	Sensitive
CircSMARCA5 [18]	Cisplatin, bleomycin	Sensitive
CircUBAP2 [52]	Cisplatin	Resistant
CircFAT1 [53]	Oxaliplatin	Resistant
CircCDR1 [54]	5-FU	Resistant
CircNCOR1 [55], circABCC1 [56], circADAM9 [57]	Radiotherapy	Resistant
CircPVT1 [58], circSFMBT2 [59], circTRIM28 [60], circCDK1 [61], circUBE2D2 [62]	Tamoxifen	Resistant
Circ_0025202 [63]	Tamoxifen	Sensitive
CircCDYL2 [64], circBGN [65], circ_0001598 [66]	Trastuzumab	Resistant
CircMMP11 [67]	Lapatinib	Resistant
CircFGFR4 [68]	Pembrolizumab	Resistant

### Radiotherapy

Radiotherapy is a standard treatment for breast cancer patients. However, radiation resistance could lead to relapse after treatment. Investigating the underlying mechanisms of breast cancer radiation resistance may help improve the outcome of patients [71]. The capacities of circRNAs to modulate breast cancer radiosensitivity have been discussed in a few studies. Overexpression of circNCOR1 reduced breast cancer radiosensitivity to promote breast cancer growth by acting as a decoy of hsa-miR-638 to regulate the level of CDK2 [55]. Circ-ABCC1 is upregulated in radio-resistant breast cancer and could induce radioresistance through acting as a decoy of miR-627-5p to increase the level of ABCC1 [56]. Circ-ADAM9 is upregulated in breast cancer, and inhibition of circ-ADAM9 could induce breast cancer radiosensitivity and apoptosis by acting as a decoy of miR-383-5p to regulate the level of PFN2 [57]. The above studies indicated that circRNAs have important functions in radiation resistance in breast cancer. However, further explorations are necessary to determine the mechanism and potential clinical value of circRNAs in breast cancer radiotherapy.

### Endocrine therapy

Endocrine therapy is a basic therapeutic strategy for ERα-positive breast cancer. However, resistance to endocrine therapy is a great obstacle in breast cancer treatment, as approximately 20%–50% of patients develop resistance to endocrine therapy [72]. Increasing studies have claimed that circRNAs have important functions in endocrine resistance and that they are considered predictive biomarkers to monitor the response to endocrine therapy. Moreover, they have great potential as therapeutic targets to overcome resistance to endocrine therapy [73]. CircPVT1 is elevated in ERα-positive breast cancer and could promote resistance to endocrine therapy by sponging miR-181a-2-3p to stabilize the expression of ESR1 and promote the activity of estrogen/ERα target genes, while targeting circPVT1 could resensitize breast cancer cells to tamoxifen treatment [58]. Circ-SFMBT2 is a crucial regulator of the ERα pathway, and overexpression of circ-SFMBT2 can induce tamoxifen resistance by enhancing ERα stability. Mechanistically, circ-SFMBT2 promotes the recruitment of RNF181 to ERα to suppress ERα ubiquitination, leading to enhanced ERα stability and elevated expression of ERα target genes. Inhibiting circ-SFMBT2 might be a potential therapeutic strategy in ER-positive breast cancer [59]. CircTRIM28 is overexpressed in breast cancer. Silencing circTRIM28 could enhance breast cancer tamoxifen sensitivity by acting as a decoy of miR-409-3p to increase the level of HMGA2 [60]. CircCDK1 is upregulated in tamoxifen-resistant breast cancer. Knockdown of circCDK1 could restore breast cancer sensitivity to tamoxifen by modulating miR-489-3p/CDK1 signaling [61]. Circ_UBE2D2 is upregulated in tamoxifen-resistant breast cancer, and circ_UBE2D2 could induce breast cancer tamoxifen resistance by regulating miR-200a-3p [62]. Hsa_circ_0025202 levels are decreased in breast cancer *vs*. control samples, and overexpression of hsa_circ_0025202 could enhance breast cancer tamoxifen sensitivity by modulating miR-182-5p/FOXO3a signaling [63]. Taken together, the above results indicate that circRNAs are crucial players in endocrine therapy of breast cancer and could serve as promising biomarkers as well as targets for ERα-positive breast cancer.

### Targeted therapy

Great developments in targeted therapies have occurred in recent decades, and the number of treatment options for breast cancer patients has increased significantly [74,75]. For HER2-positive breast cancer, trastuzumab is the main molecularly targeted agent. However, approximately 25% of patients develop resistance to trastuzumab, indicated by rapid recurrence. The clinical efficacy could be improved by overcoming drug resistance. CircRNAs have been reported to be correlated with breast cancer targeted therapy resistance. CircCDYL2 is a promising biomarker for trastuzumab resistance and has vital functions in resistance against trastuzumab, and patients with elevated levels of circCDYL2 exhibit rapid recurrence. Mechanistically, circCDYL2 prevents GRB7 degradation to promote GRB7 interaction with FAK and sustains AKT and ERK1/2 activities to sustain the HER2 pathway [64]. Circ-BGN is overexpressed in breast cancer and contributes to trastuzumab resistance by suppressing cell ferroptosis through inducing deubiquitination of SLC7A11 mediated by OTUB1, leading to poor OS. Inhibiting circ-BGN could restore sensitivity against trastuzumab in breast cancer cells [65]. Circ_0001598 levels were elevated in breast cancer and modulated trastuzumab resistance in HER2-positive breast cancer by regulating the miR-1184/PD-L1 axis [66]. Circ-MMP11 is upregulated in lapatinib-resistant breast cancer. Overexpression of circ-MMP11 could induce resistance against lapatinib in breast cancer through acting as a decoy of miR-153-3p and regulating the level of ANLN [67]. The above findings showed that circRNAs play key roles in breast cancer resistance to targeted therapy, and further exploration is warranted to transform these findings to clinical application for breast cancer.

### Immunotherapy

Recently, increasing attention has been given to breast cancer immunotherapy, which could help improve the response of breast cancer to other therapies [76,77]. However, not every patient can benefit from immunotherapy. Therefore, predictive biomarkers are needed to better select patients who may respond to immunotherapy [78]. CircRNAs have great capacities in immune evasion of a series of cancers, including breast cancer. However, the potential mechanisms of circRNAs in the immunotherapy resistance of breast cancer are mostly unclear and need to be further explored. CircFGFR4 is overexpressed in TNBC. CircFGFR4 acts as a decoy of miR-185-5p and increases the expression of CXCR4, so overexpression of circFGFR4 led to reduced infiltration of CD8+ T cells and enhanced resistance against anti-PD-1 immunotherapy in TNBC [68]. The circHIPK3 level is elevated and associated with a lower immunotherapy response in breast cancer [79]. The above findings indicate that circRNAs have great potential as predictive biomarkers for immunotherapy response as well as immunotherapeutic targets in breast cancer.

### CircRNAs in breast cancer prognosis

CircRNAs have been identified as promising prognostic biomarkers in breast cancer [80,81]. The increased circCDYL level in breast cancer serum and tissue is associated with shorter survival, indicating the possibility of circCDYL as a predictive biomarker for breast cancer prognosis [82]. Hsa_circ_0067842 is elevated in breast cancer and is related to worse breast cancer outcomes [83]. The hsa_circ_0001785 level in plasma is closely associated with breast cancer histological grade, TNM stage and distant metastasis, indicating the potential diagnostic and predictive capacity of hsa_circ_0001785 in breast cancer plasma [31]. CircCNOT2 is detectable in plasma and is correlated with poor progression-free survival (PFS) in advanced breast cancer patients with aromatase inhibitor (AI) therapy [84]. Plasma hsa_circ_0008673 levels are related to shorter overall survival (OS) and disease-specific survival (DSS) in breast cancer, indicating that it acts as a promising predicator for breast cancer prognosis evaluation [21]. The serum circRASSF2 level is associated with worse OS and PFS in breast cancer patients [22]. A high level of circBCBM1 in breast cancer is related to poorer brain metastasis-free survival, so circBCBM1 could act as a promising prognostic biomarker in breast cancer [24]. Collectively, the above studies indicated that circRNAs may act as promising prognostic biomarkers for breast cancer patients (Table 3).

**Table 3:** CircRNAs in breast cancer prognosis

Name	Dysregulation	Prognosis	Source
CircCDYL [82]	Upregulated	Worse	Serum, tissue
Hsa_circ_0067842 [83]	Upregulated	Worse	Tissue
Hsa_circ_0001785 [31]	Upregulated	Worse	Plasma
CircCNOT2 [84]	Upregulated	Worse	Plasma
Hsa_circ_0008673 [21]	Upregulated	Worse	Plasma
CircRASSF2 [22]	Upregulated	Worse	Serum
CircBCBM1 [24]	Upregulated	Worse	Serum

## Conclusions

Recently, circRNA has become a hot topic in cancer research. CircRNA dysregulation has been discovered in a variety of malignancies, and circRNAs have important functions in tumorigenesis as well as cancer progression through diverse molecular mechanisms [85,86]. Recent studies have claimed that various circRNAs are related to breast cancer tumorigenesis, recurrence and multidrug resistance through various mechanisms [87–89]. In addition, circRNAs are crucial players in treatment resistance and are correlated with the clinicopathological characteristics of breast cancer patients. Here, we summarized recent studies on the characteristics and roles of circRNAs in breast cancer.

An increasing number of studies have indicated that circRNAs could act as promising diagnostic and prognostic biomarkers. Indeed, tissue-specific expression and resistance to exonucleases make circRNAs an attractive new class of noninvasive biomarkers for clinical application [90]. However, these studies have some limitations, such as inconsistency and nonreproducibility. Most of the studies published are based on breast cancer cell lines or tissue samples from rather small populations, and most lack validation in larger independent cohorts. Therefore, a larger number of samples are needed to confirm the results. Due to sample heterogeneity, differences in detection methods and a lack of proper validation techniques, most assessed circRNAs are not sensitive or specific enough to be used as clinical biomarkers. Currently, the prospective value of circRNAs as biomarkers is under investigation in clinical trials in a series of human malignancies, including breast cancer. For instance, serum hsa_circ_0001785 and hsa_circ_100219 have been investigated as promising diagnostic or prognostic biomarkers in breast cancer (NCT05771337).

In spite of the advances in breast cancer treatment strategies, development of resistance to these therapeutics is still an obstacle in clinical setting [91,92]. The roles and underlying mechanisms of circRNAs in predicting breast cancer treatment responsiveness have been investigated [93]. However, most of the studies have focused on chemotherapy and endocrine therapy, and there are still not enough studies covering the roles and possible mechanisms of circRNAs in radiotherapy, immunotherapy or targeted therapy in breast cancer, which is worth further exploration. In addition, there is still no circRNA drug that can be used in clinical settings due to low specificity and instability. More investigations are needed to promote the application of circRNAs as targetable molecules in future clinical treatment.

In conclusion, circRNAs have important functions in the initiation and progression of breast cancer. Understanding the mechanisms by which circRNAs function would aid in the development of novel circRNA-based therapeutic strategies for breast cancer treatment. Further explorations, especially high-quality animal studies and large-scale clinical trials, are needed to transform basic research into clinical applications.

## Data Availability

Data will be made available on request.

## Abbreviations

circRNA: Circular RNA
ncRNA: Noncoding RNA
ceRNA: Competitive endogenous RNA
TNBC: Triple-negative breast cancer
ADM: Adriamycin
PTX: Paclitaxel
DDP: Cisplatin
5-FU: 5-fluorouracil
PFS: Progression-free survival
OS: Overall survival
DSS: Disease-specific survival
